# p53-loss induced prostatic epithelial cell plasticity and invasion is driven by a crosstalk with the tumor microenvironment

**DOI:** 10.1038/s41419-025-07361-1

**Published:** 2025-01-26

**Authors:** Darya Yanushko, Beatriz German Falcon, Rana El Bizri, Despoina Pervizou, Robin Dolgos, Céline Keime, Tao Ye, Christelle Thibault-Carpentier, Clementine Le Magnen, Sandrine Henri, Gilles Laverny, Daniel Metzger

**Affiliations:** 1 Institut de Génétique et de Biologie Moléculaire et Cellulaire, Illkirch, France; 2 Centre National de la Recherche Scientifique (CNRS), Illkirch, France; 3 Institut National de la Santé et de la Recherche Médicale (INSERM), Illkirch, France; 4 Université de Strasbourg, Strasbourg, France; 5 Centre d’Immunologie de Marseille-Luminy, Aix Marseille Université, INSERM, CNRS, Marseille, France; 6 Institute of Medical Genetics and Pathology, Department of Urology, Department of Biomedicine, University Hospital Basel, Basel, Switzerland; 7 Present Address: Center for Prostate Disease Research, Murtha Cancer Center Research Program, Departement of Surgery, Walter Reed Army Medical Center and Uniformed University of the Health Sciences, The Henry M. Jackson Foundation for the Advancement of Military Medicine, Bethesda, MD USA; 8 Present Address: Bellvitge Institute for Biomedical Research (IDIBELL), Barcelona, Spain

**Keywords:** Prostate cancer, Cancer microenvironment

## Abstract

Prostate cancer is a heterogeneous disease with a slow progression and a highly variable clinical outcome. The tumor suppressor genes *PTEN* and *TP53* are frequently mutated in prostate cancer and are predictive of early metastatic dissemination and unfavorable patient outcomes. The progression of solid tumors to metastasis is often associated with increased cell plasticity, but the complex events underlying *TP53*-loss-induced disease aggressiveness remain incompletely understood. Using genetically engineered mice, we show that *Trp53* deficiency in *Pten*-null prostatic epithelial cells (PECs) does not impact early cell proliferation and neoplasia formation, nor growth arrest and senescence entry at a later time. However, *Trp53*-deficiency enhances invasive adenocarcinoma development and promotes metastatic cell dissemination. Importantly, our single-cell transcriptomic and chromatin accessibility analyses combined with histological examinations uncovered an epithelial cell population characterized by an induction of Jak/Stat3 signaling and displaying mesenchymal features. Moreover, we show that the transcriptomic signature of this cell population is prominent in tumors of patients with high-risk prostate cancer or metastatic disease. In addition, our in vivo and organoid-based experiments provide evidence that PEC plasticity occurs through bi-directional communication with cancer-associated fibroblasts (CAFs). Thus, our study demonstrates that p53 loss induces a protumorigenic crosstalk between PECs and CAFs, and identifies new vulnerabilities that might be targeted to limit cancer progression.

## Introduction

Prostate cancer is the most commonly diagnosed non-cutaneous male malignancy and the second cause of cancer-related death in Western countries [1]. It is a heterogeneous disease with a slow progression and a highly variable clinical outcome. The tumor suppressors *PTEN* and *TP53* are among the most frequently altered genes in prostate cancer [2]. *PTEN* mutations have been identified in 10–15% of all prostate tumors and in up to 60% of advanced prostate cancers [3, 4]. Moreover, *TP53* mutations are present in up to 75% of advanced tumors and are highly associated with metastatic disease [2, 3]. However, there is emerging evidence that *TP53* mutations are already present at a high frequency in primary prostatic tumors and are predictive of early metastatic dissemination and an unfavorable patient outcome [5, 6]. Though, the molecular and cellular mechanisms underlying the progression of indolent PTEN- and p53-deficient tumors to aggressive and metastatic cancer remain elusive.

Over the recent years, single-cell analyses uncovered a large cellular heterogeneity of localized prostatic tumors [7–9]. While tumorigenesis initiates in prostatic epithelial cells, various cell types, including cancer associated fibroblasts (CAF), immune and endothelial cells produce extra-cellular matrix and secrete signaling molecules that might exert pro-tumorigenic effects, and thus contribute to tumor progression [10, 11]. However, the complex communication network during tumor progression remains poorly characterized.

We have previously shown that Pten^(i)pe−/−^ mice, in which *Pten* is inactivated in prostatic luminal cells at adulthood, develop prostatic intraepithelial neoplasia (PIN) due to enhanced proliferation of prostatic epithelial cells (PECs), featuring stabilization of p53, followed by a progressive growth arrest with characteristics of cell senescence. PIN formation and progression is also characterized by HIF1 signaling activation and a switch from Luminal-A to Luminal-C epithelial cell state [12]. Some PINs progressed to adenocarcinoma, but no metastases were observed [13, 14]. Moreover, Chen et al. reported that Pb-Cre4-mediated inactivation of *Pten* and *Trp53* in prostatic epithelial cells induces more aggressive tumors with no signs of senescence [15].

To unravel the mechanisms underlying increased aggressiveness induced by *Trp53* loss, we performed detailed longitudinal studies of Pten/Trp53^(i)pe−/−^ mice, in which *Pten* and *Trp53* are selectively inactivated in prostatic luminal cells at adulthood. Our results show that p53 deficiency does not impact the early proliferation of *Pten*-null epithelial cells and PIN formation, nor growth arrest and senescence entry at a later time. However, it induces epithelial cell plasticity, enhances adenocarcinoma formation and promotes metastatic dissemination. Importantly, we unravel that epithelial cell plasticity is induced in a non-cell-autonomous manner by a feedforward loop stimulating IL-6 production by CAFs, which in turn enhances Jak/Stat3 signaling in epithelial cells. In addition, aggressive and metastatic prostate tumors in patients are characterized by a high score of the transcriptomic signature identified in PECs of Pten/Trp53^(i)pe−/−^ mice. Thus, p53 loss promotes prostate cancer progression by inducing a pro-tumorigenic crosstalk between *PTEN*-deficient prostatic epithelial cells and cancer-associated fibroblasts.

## Materials and methods

### Study design

The objective of the study was to identify cell-intrinsic and extrinsic factors driving prostate cancer progression. To this aim, we generated cohorts of Pten/Trp53^(i)pe−/−^ and Pten^(i)pe−/−^ mice and analyzed them at multiple time points. Only male mice were analyzed, as prostate cancer modeling is relevant only in animals of this sex. Investigators were not blinded to animals’ genotypes. The impact of combined *Pten* and *Trp53* loss in luminal cells on prostate weight, histology, and cell proliferation was determined by three investigators. Sample size for animal experiments was estimated using EDA (NC3R), assuming 10% of interindividual variability and 20% of the observed effect. No outliers were excluded. In vitro results are representative of three biological replicates obtained from independent experiments.

### Mice

Pten^(i)pe−/−^ and Pten/Trp53^(i)pe−/−^ male mice, as well as sex-matched Pten^L2/L2^ and Pten/Trp53^L2/L2^ control littermates (all on a C57BL/6 genetic background) were generated by intraperitoneal injection of Tamoxifen (1 mg/mouse/day) for 5 days to 8 to 10 week old PSA-CreER^T2(Tg/0)^/Pten^L2/L2^, PSA-CreER^T2(Tg/0)^/Pten^L2/L2^/Trp53^L2/L2^, PSA-CreER^T2(0/0)^/Pten^L2/L2^ and PSA-CreER^T2(0/0)^/Pten^L2/L2^/Trp53^L2/L2^ mice, respectively, as described [13, 14]. Mice breeding and maintenance were done in the accredited IGBMC/ICS animal house (D-67-218-37), in compliance with French and EU regulations on the use of laboratory animals for research.

### Histological examination

Five µm histological sections were prepared from paraffin-embedded tissues and dried overnight at 37 °C. Hematoxylin and eosin (HE) staining was performed according to standard protocols. Slides were scanned in brightfield mode with a NanoZoomer digital slide scanner (Hamamatsu) and analyzed with the NDP.view2 Viewing software (Hamamatsu). Representative images are provided.

### Immunostaining of the tissue sections

Five µm paraffin tissue sections were deparaffinized according to standard protocols and incubated for 20 min in SignalStain Citrate Unmasking Solution (10X) (CST 14746) in a pressure cooker for heat-induced antigen retrieval. The following primary antibodies were used: p53 (CM5) (Leica, P53-CM5P-L; 1:200), p21 (BD Pharmingen, 556430, 1:50), pAkt (S473) (D9E) XP (CST, 5315S; 1:200), Trop2 (R&D Systems, AF1122; 1:50), Ki-67 (Thermo Fisher Scientific, MA5-14520; 1:200), E-cad (CST, 14472S; 1:200), Vim (CST, 5741S; 1:200), pStat3 (Tyr705) (D3A7) XP (CST, 9145S; 1:200), CD45 (Abcam, ab10558; 1:200), p16 [EPR20418] (Abcam, ab211542; 1:600) and pan-keratin (Proteintech, 26411-1-AP; 1:1500).

For immunohistochemistry, one drop of SignalStain® Boost IHC Detection Reagent (CST 8114) was added to each section, and SignalStain® DAB Substrate Kit (CST 8059) used to develop the signal, according to the manufacturer’s instructions. The sections were counterstained with hematoxylin and mounted. Images were acquired using a NanoZoomer digital slide scanner (Hamamatsu) and analyzed with the NDP.view2 Viewing software (Hamamatsu).

For immunofluorescence, secondary antibodies coupled to Alexa Fluor 555 (Invitrogen, A31570; 1:400), Alexa Fluor 647 (Invitrogen, A32849; 1:400) or Alexa Fluor 488 (Invitrogen, A21206; 1:400) were used. The sections were mounted with Fluoromount-G^TM^ (Invitrogen, 00-4959-52). Images were acquired with a Leica DM 4000 B microscope equipped with a Photometrics CoolSNAP HQ2 camera.

The number of Ki-67-positive, p16-positive, and pStat3-positive cells was determined with the QuPath software [16].

### SA-ß-gal staining

SA-ß-gal staining was performed on 10 µm frozen prostate sections using the senescence ß-Galactosidase Staining Kit (CST 9860), following the manufacturer’s instructions. Sections were counterstained with hematoxylin.

### Flow cytometry

To distinguish circulating cells from tissue-resident cells, mice were injected intravenously with 3 μg of anti CD45.2-FITC 3 min before euthanasia. Prostates were collected, the DLP isolated, weighted, cut into small pieces and incubated for 20 min at room temperature with type II collagenase (Worthington Biochemical) and DNase I (Sigma) in 2% FCS RPMI under frequent pipetting. The enzymatic reaction was stopped by addition of 2% v/v of 0.5 M EDTA. The cellular suspension was passed through a 5 ml syringe with 18 G needle for complete tissue dissociation. The single-cell solution was filtered through a 70 μm cell strainer and centrifuged. The pellet was resuspended in PBS, 5 mM EDTA, 2% FCS and was re-filtered through a 30 μm cell strainer (Miltenyi).

Cells were preincubated with anti-Fc receptor antibody (clone 2.4G2) and stained with appropriate antibodies in PBS-5 mM EDTA, 2% FCS at 4 °C for 30 min at dark. Viability was assessed with Zombie UV™ Fixable Viability dye (Biolegend). Flow cytometry was performed on FACS Symphony or Fortessa X20 systems (BD Biosciences) and data analyzed with Flowjo software (Flowjo LLC). Doublets and dead cells were excluded from the analysis. For the analysis of myeloid cells (neutrophils and macrophages), B, T and NK cells were gated out using a Lin^–^ gate (CD5^–^, CD19^–^, CD161c^–^). Antibodies used are listed in Supplementary Table 9. The gating strategies for lymphoid and myeloid cells are presented in Fig. S5. Absolute numbers were estimated using CountBright™ absolute count microbeads for flow cytometry (Invitrogen) and normalized by the prostate weight.

### scRNA-seq analysis

Mouse prostates were dissociated into single cells as described [12]. Cells from 3 prostates were pooled, stained with DAPI and living cells (DAPI-negative) were FACS-sorted using a BD FACSAria^TM^ Fusion flow cytometer. Trypan blue exclusion assay was used to determine the cell number and viability of FACS-sorted cells using a Neubauer Chamber. Samples with a cell viability of >95% were processed with a Chromium Controller (10X Genomics, Leiden, The Netherlands). Sixteen thousand cells were loaded per well in nanoliter-scale Gel Beads-in-Emulsion (GEMs). Single-cell 3′ mRNA-seq libraries were generated according to “User guide Chromium Next GEM Single Cell 3 prime Reagent Kits v3.1 (Dual index) (10X Genomics, PN CG000315 - Rev.D). Briefly, cells were partitioned into droplets with barcoded gel beads and reverse transcription master mix using Chromium Chip G. After complementary DNA (cDNA) synthesis and barcoding from poly-adenylated RNA, GEMs were disrupted and pooled before cDNA amplification by 11 polymerase chain reaction (PCR) cycles. Following enzymatic fragmentation and size selection of cDNA amplicons, sequencing libraries were constructed by adding Illumina P5 and P7 adapters (San Diego, USA) and i5 and i7 sample indexes by end repair, A-tailing, adaptor ligation, and 10 cycles of PCR amplification. Quality control and quantification of libraries was performed with a Bioanalyzer 2100 (Agilent Technologies, Santa Clara, CA). The generated library was sequenced on Illumina HiSeq 4000 as 100 bases paired-end reads and Illumina NextSeq 550 as 28 + 100 bases paired-end reads. RTA 2.7.7, bcl2fastq 2.20.0.422 and Cell Ranger 3.0.2 mkfastq were used for image analysis, base calling and demultiplexing. Cell Ranger 7.0.0 count was used for alignment, barcode and UMI (Unique Molecular Identifier) filtering and counting, with a reference created with Cell Ranger 7.0.0 mkref using GRCm39 assembly of Mus musculus genome, Ensembl release 107 annotations. The Read10X function of the Seurat version (v) 4.3 (R v 4.2.2) was used to read the output of the Cell Ranger pipeline and obtain a matrix of the number of UMI of each gene detected in each cell.

### snATAC-seq analysis

Mouse DLPs were dissociated into single cells as described [12]. Nuclei were isolated from 800 000 cells by applying Nuclei isolation buffer (10 mM Tris-HCl pH 7.4, 10 mM NaCl, 3 mM MgCl_2_, 0.1% Tween-20, 0.1% IGEPAL CA-630, 0.01% Digitonin, 1% BSA) for 5 min and washed according to the 10X Demonstrated Protocol (CG000169). For each sample, 16,000 nuclei were loaded on a 10X Chromium Controller (10X Genomics, Leiden, The Netherlands) to recover 10,000 nuclei.

Chromium Next GEM Single Cell ATAC Reagent Kits v1.1 (10X Genomics ref. CG000209 Rev C) were used to generate single-nuclei libraries. Nuclei were fragmented and adapter sequences were added through a Transposition Mix. Gel beads with barcodes and a Master Mix were combined with the transposed nuclei on a Chromium Chip H to generate GEMs, which were then broken and pooled to produce barcoded single-stranded DNA. The leftover biochemical reagents and unused barcodes were removed from the reaction mixture using magnetic and SPRI beads. Library construction was performed via PCR with the addition of P7 and a sample index. Quality control and quantification of libraries were performed using a Bioanalyzer 2100 (Agilent Technologies, Santa Clara, CA). Generated libraries were sequenced on Illumina HiSeq 4000 and NextSeq 2000 sequencers as 100 bases paired-end reads. Cell Ranger ATAC 2.0.0 and the mouse reference cellranger-arc-mm10-2020-A-2.0.0 were used for demultiplexing, alignment, barcode filtering and counting. The Read10X function of the Seurat v 4.3.0 (R v 4.2.2) was used to read the output of the Cell Ranger pipeline and to obtain a matrix of the number of reads of each peak detected in each cell.

Reads from barcodes of the same cell type were pooled using Signac 1.9.0. Cell type-specific peak calling was performed using macs2 2.2.7.1. Peaks were annotated to the nearest gene using Homer software mm10 genome ucsc v6.4. eRegulons were determined using the Scenic+ package (version 0.1.dev456 + g9662363).

### Culture and analysis of PEC-derived organoids and cancer-associated fibroblasts

Mouse DLPs were dissociated into single cells, as described [12]. For fibroblast culture, single-cell suspensions obtained from DLPs of Pten^(i)pe−/−^ and Pten/Trp53^(i)pe−/−^ mice 5 months AGI were seeded in DMEM [4.5 g/L glucose, 20% FCS, 1% penicillin/streptomycin] at 37 °C and 5% CO_2_. After 1 passage, only fibroblasts were maintained in culture. At 70% confluence, the media was replaced, collected 48 h later, and centrifuged for 5 min at 400 g.

Pten^−/−^ and Pten/Trp53^−/−^ organoid cultures were established from DLPs of Pten^(i)pe−/−^ and Pten/Trp53^(i)pe−/−^ mice 3 months AGI, respectively, as described [12, 17]. 5000 cells were seeded per Cultrex dome (R&D systems, 3533-010-02) and after 1 week, the culture media was replaced by conditioned medium from CAFs of Pten^(i)pe−/−^ or Pten/Trp53^(i)pe−/−^ mice, with or without Ruxolitinib (10 µM) (MedChemExpress, HY-50856), with control IgG (R&D Systems; AB-108) or IL-6 neutralizing antibody (R&D Systems; AB-406-NA), or with “organoid medium” supplemented with IL-6 (at a final concentration of 2 or 4 ng/mL) (PeproTech, 216-16) for 48 h.

To perform histological analyses of murine organoids, matrigel domes were digested for 1 h at 37 °C in dispase (2 mg/mL) (StemCell Technologies, 07913). Organoids were fixed in 10% formalin for 24 h, included in HistoGel™ (Epredia, HG-4000-012), embedded in paraffin and processed for HE staining or immunofluorescent detections following standard protocols.

### IL-6 level determination

The culture media of organoids and fibroblasts were replaced 1 week after seeding with cognate culture media, collected 48 h later, centrifuged at 400 g for 5 min and supernatants were collected. IL-6 levels were determined with the Mouse IL-6 Uncoated ELISA Kit (Invitrogen, 88-7064-88) in 96-well plates (Corning, 9018), according to manufacturer’s protocol.

### Culture and analysis of patient-derived organoids P20-11

P20-11 patient-derived organoids (PDO) were established from a lung metastasis specimen (HSPC patient)[18]. PDO-xenografts (PDOXs) were obtained by subcutaneous injection of dissociated PDO cells in NSG male mice, with testosterone pellet. PDOX tumors were then dissociated and cultured as organoids (PDOXOs). PDOXOs were passaged to single-cell suspension and allowed to recover for five days. Organoids were then treated with 4 ng/mL IL-6 or vehicle for 5 days. Organoid collection, inclusion and staining were performed as previously reported [18, 19]. For immunofluorescence, primary antibodies against Vimentin (Abcam, ab8978, 1:2000) and E-Cadherin (CST, 3195, 1:500) and secondary antibodies coupled to Alexa Fluor 555 (Invitrogen, AB_2535849; 1:1000) and Alexa Fluor 647 (Invitrogen, AB_2535806; 1:1000) were used.

### EMTc signature and human dataset analyses

The EMTc signature was generated by selecting the top 25 globally distinguishing genes of the EMTc cluster with the lowest adjusted *p*-value and a log2 fold change > 1 in the scRNAseq data. This list of genes was then refined by selecting the 9 genes up-regulated in transcriptomes of tumor tissues versus healthy tissues from the TCGA PRAD cohort.

The correlation of EMTc signature with the *TP53* status and the Gleason score was analyzed in the TCGA PRAD cohort (*n* = 496 patients with prostate cancer) and in the cohort of Grasso et al. (*n* = 76 patients with prostate cancer) [20]. The score corresponds to the mean of the quartiles for each individual gene.

### Data analysis

Statistical analysis was performed using the GraphPad Software Prism 9.

## Results

### Prostatic epithelial cells of Pten/Trp53^(i)pe−/−^ mice become senescent after a proliferation phase

To determine the impact of p53 deficiency in *Pten*-null PECs, we compared prostate tumorigenesis of Pten^(i)pe−/−^ and Pten/Trp53^(i)pe−/−^ mice. Most PECs of both mouse lines were phosphorylated AKT (S473) positive in PINs 3 months after gene inactivation (AGI), and expressed TROP2, a marker of Luminal-C cells (Supplementary Fig. 1A and B). In contrast, p53 protein was detected in the nuclei of PECs of Pten^(i)pe−/−^ mice, but not in those of Pten/Trp53^(i)pe−/−^ mice (Supplementary Fig. 1B), demonstrating efficient inactivation of *Pten* and *Trp53* in prostatic luminal cells of Pten/Trp53^(i)pe−/−^ mice.

**Fig. 1:**
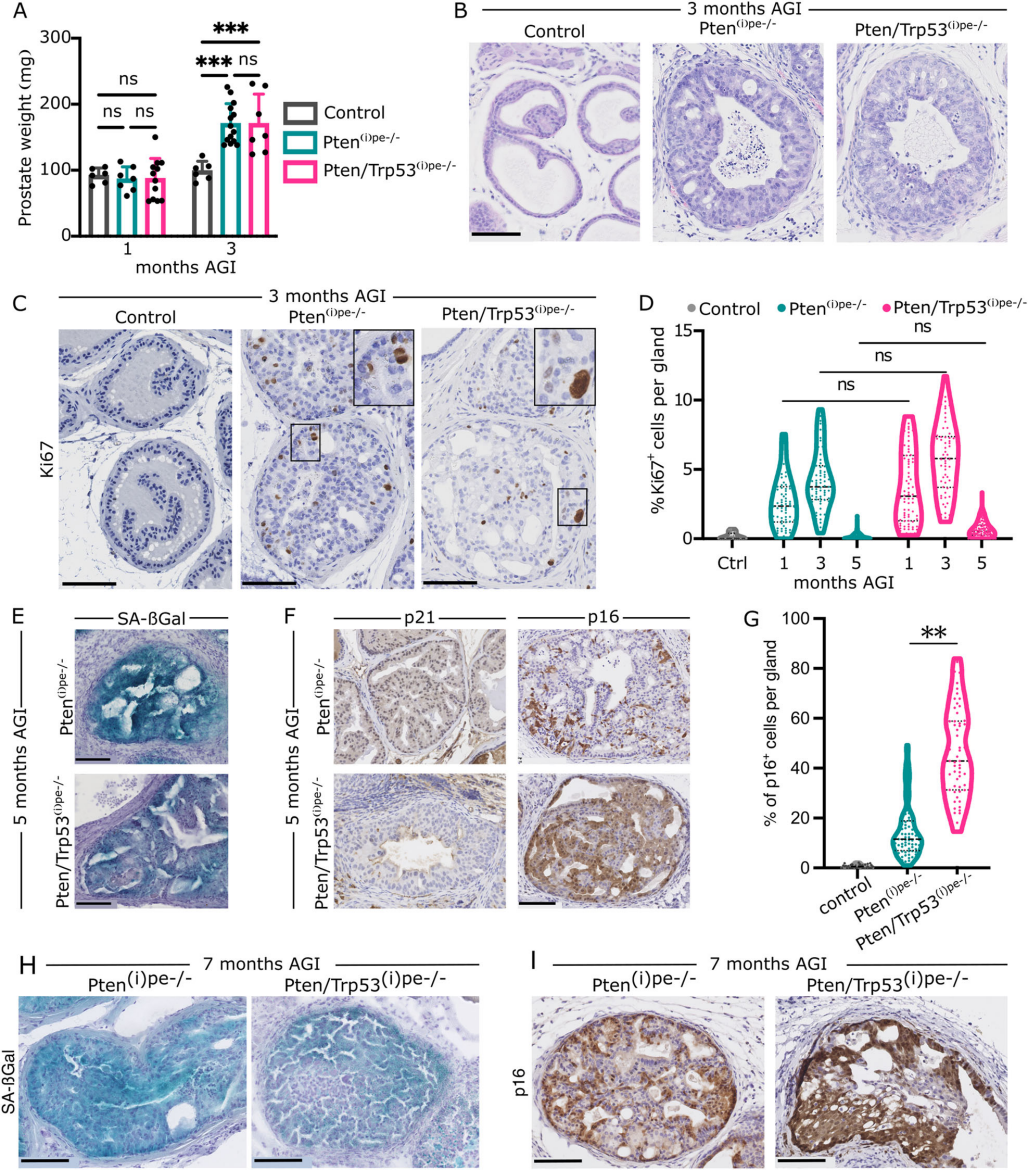
Characterization of early-stage prostate tumors in Pten/Trp53^(i)pe−/−^ mice. **A** Prostate weight of control (Pten^L2/L2^ and Pten/Trp53^L2/L2^ mice), Pten^(i)pe−/−^ and Pten/Trp53^(i)pe−/−^ mice 1 and 3 months AGI. *n* ≥ 6 per group. ns non-significant, *p* ≥ 0.05; ***p < 0.001. Two-way ANOVA followed by Tukey’s post-hoc test. **B** Representative hematoxylin-eosin (HE) staining of DLP sections from control, Pten^(i)pe−/−^ and Pten/Trp53^(i)pe−/−^ mice, 3 months AGI. Scale bar: 100 µm. *n* ≥ 6 per group. **C**, **D** Representative Ki-67 staining (brown) of DLP sections from control, Pten^(i)pe−/−^ and Pten/Trp53^(i)pe−/−^ mice 3 months AGI (**C**) and quantification of Ki-67 positive PECs per gland at 1, 3 and 5 months AGI (**D**). *n* ≥ 3 mice per group. ns non-significant, *p* ≥ 0.05. Two-way ANOVA followed by Tukey’s post-hoc test. Scale bar: 100 µm. **E** Representative Senescence-associated ß-galactosidase (SA-ßGal) staining (blue) of DLP sections from Pten^(i)pe−/−^ and Pten/Trp53^(i)pe−/−^ mice 5 months AGI. Scale bar: 100 µm. *n* = 3 mice per group. **F**, **G** Representative immunohistochemical staining of p21 and p16 (brown) (**F**) and quantification of p16 positive (+) PEC per gland (**G**) in DLP of control, Pten^(i)pe−/−^ and Pten/Trp53^(i)pe−/−^ mice 5 months AGI. Scale bar: 100 µm. *n* ≥ 3 per group. **p < 0.01; One-way ANOVA with post-hoc Tukey. **H** Representative SA-ßGal staining (blue) of DLP sections from Pten^(i)pe−/−^ and Pten/Trp53^(i)pe−/−^ mice 7 months AGI. Scale bar: 100 µm. *n* = 3 mice per group. **I** Representative immunohistochemical staining of p16 (brown) in DLP of Pten^(i)pe−/−^ and Pten/Trp53^(i)pe−/−^ mice 7 months AGI. Scale bar: 100 µm. *n* ≥ 3 per group.

The average prostate weight of control mice, as well as of Pten^(i)pe−/−^ and Pten/Trp53^(i)pe−/−^ mice 1 month AGI, was around 100 mg, and increased to 180 mg in both mutant mice 3 months AGI, but not in controls (Fig. 1A). Hematoxylin and eosin (HE) staining of prostatic sections showed the presence of PINs and a stromal reaction in dorso-lateral prostates (DLPs) of both mutant mice 3 months AGI (Fig. 1B). The proliferation rate was 2.5-fold higher in PINs of the DLP of mutant mice 1 month AGI than in PEC of aged-matched control mice, and further increased 3 months AGI. However, it was below 1% in control and mutant mice 5 months AGI (Fig. 1C, D), and PINs of both Pten^(i)pe−/−^ and Pten/Trp53^(i)pe−/−^ mice were positive for senescence-associated β-galactosidase (SA-βGal), a faithful marker of cell senescence [21] (Fig. 1E, Supplementary Fig. 1C). Moreover, while most PECs of Pten^(i)pe−/−^ mice expressed the cyclin-dependent kinase inhibitor p21, no p21-positive cells were detected in PINs of Pten/Trp53^(i)pe−/−^ mice 5 months AGI (Fig. 1F). In contrast, the proportion of p16-positive cells was 5-fold higher in DLP of Pten/Trp53^(i)pe−/−^ mice than of Pten^(i)pe−/−^ mice (Fig. 1F, G, Supplementary Fig. 1C). In addition, SA-ßgal staining and abundant p16-positive cells were still present in glands of DLP of Pten/Trp53^(i)pe−/−^ mice 7 months AGI (Fig. 1H, I). Together, these results show that p53 loss does not affect early PIN formation induced by *Pten* inactivation at adulthood, and that both *Pten-* and *Pten/Trp53*-deficient PINs in DLP become senescent.

As PECs in the anterior prostate (AP) of control, Pten^(i)pe−/−^ and Pten/Trp53^(i)pe−/−^ mice were SA-ßGal-positive 5 months AGI (Supplementary Fig. 1D), possibly due to the expression of the ß-galactosidase isoform *Glb1l3* [22], this assay could not be used to assess cell senescence in this lobe. However, some p16-positive cells were detected in TROP2-positive luminal cells in the AP of Pten/Trp53^(i)pe−/−^ mice (Supplementary Fig. 1E), indicative of senescent cells. Moreover, as PIN formation, severity and tumor progression were highly variable in the AP amongst mice and glands of a given mouse (Supplementary Fig. 1E and F), these results show that p53 loss does not prevent PECs’ senescence, and underline lobe-specific prostate tumor progression.

### Trp53 deficiency promotes PECs dissemination

**Fig. 2:**
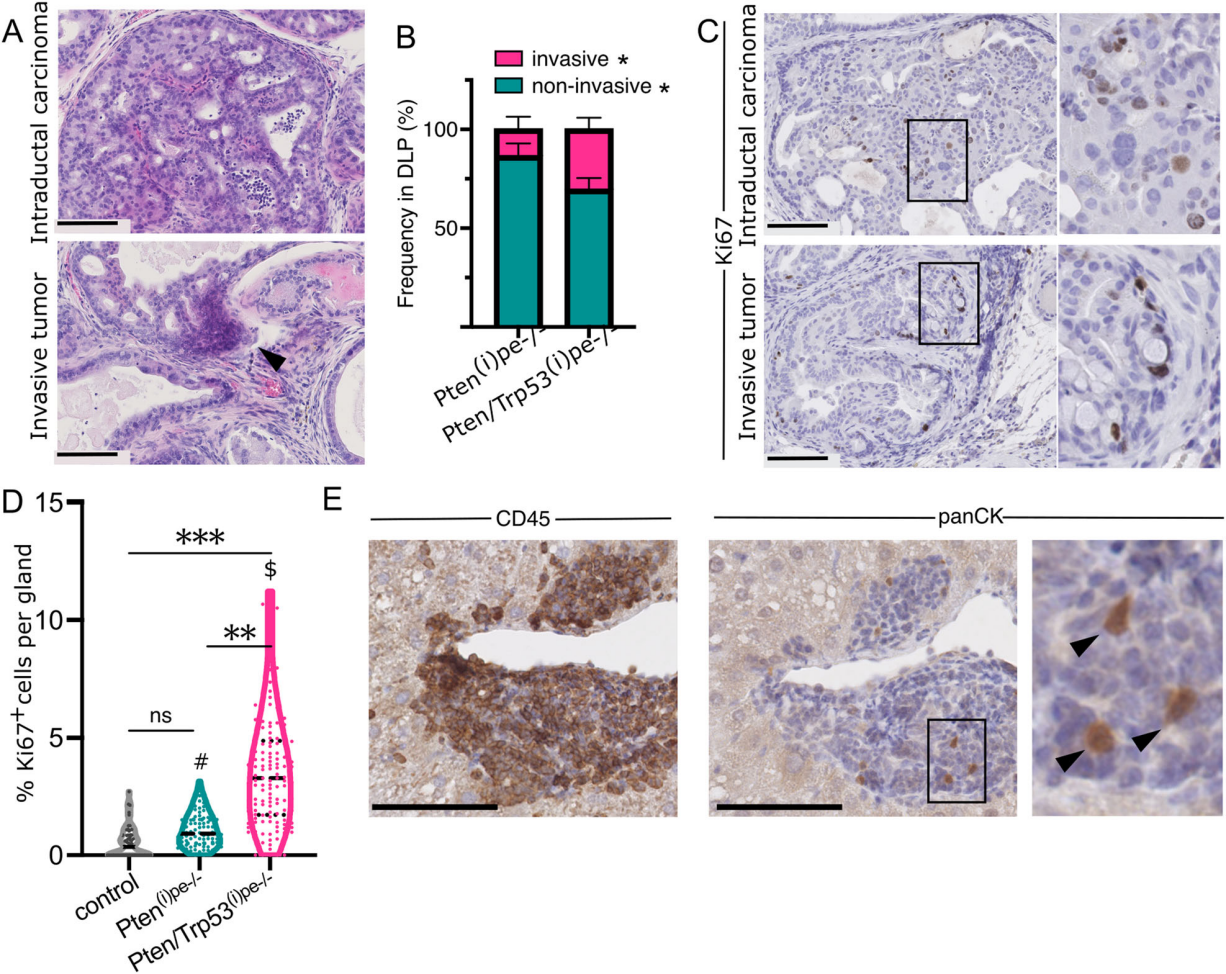
Characterization of tumor progression in Pten/Trp53^(i)pe−/−^ mice. **A** Representative HE staining of DLP of Pten/Trp53^(i)pe−/−^ mice 7 months AGI. Top panel, intraductal carcinoma (IDC); bottom panel, invasive adenocarcinoma (pointed by an arrow). Scale bar: 100 µm. *n* = 4 mice per group. **B** Frequency of invasive and non-invasive tumors in DLPs of Pten^(i)pe−/−^ and Pten/Trp53^(i)pe−/−^ mice 5–7 months AGI. *n* = 4 mice per group, *n* = 100 glands per group. Paired *t*-tests. *p < 0.05. **C** Representative Ki-67 staining of DLP sections of Pten/Trp53^(i)pe−/−^ mice 7 months AGI with IDC and invasive adenocarcinoma. *n* = 4 mice per group. Scale bar: 100 µm. **D** Quantification of Ki-67-positive PECs per gland in DLP of control, Pten^(i)pe−/−^ and Pten/Trp53^(i)pe−/−^ mice 7 months AGI. *n* ≥ 4 mice per group. 2-way ANOVA followed by Tukey’s post-hoc test. **p < 0.01; ***p < 0.001; ns non-significant, *p* ≥ 0.05; #*p* ≥ 0.05 compared to Pten^(i)pe−/−^ 5 months AGI (Fig. 1D); $*p* < 0.05 compared to Pten/Trp53^(i)pe−/−^ 5 months AGI (Fig. 1D). **E** Representative immunohistochemical detection of CD45 and pan-cytokeratin (panCK) in liver of Pten/Trp53^(i)pe−/−^ mice 6 months AGI. Scale: 100 µm. *n* = 15 mice. Arrows point to panCK^+^ cells.

Between 5 and 7 months AGI, all Pten^(i)pe−/−^ and Pten/Trp53^(i)pe−/−^ mice harbored PINs and intraductal carcinoma (IDC) within the DLPs, but invasive tumors were twice more frequent in Pten/Trp53^(i)pe−/−^ mice (Fig. 2A, B). Whereas the proliferation rate of epithelial cells in glands of Pten^(i)pe−/−^mice was below 4% and similar to that of control mice, it went up to 11% in glands of Pten/Trp53^(i)pe−/−^ mice, with an average of 4% (Fig. 2C, D). Of note, 20% of Pten/Trp53^(i)pe−/−^ mice had a prostate mass above 300 mg and developed large tumoral masses with sarcomatoid histology in the AP (Supplementary Fig. 2A & B). Importantly, Pten/Trp53^(i)pe−/−^ mice with a prostate weight below 300 mg never harbored sarcomatoid tumors, and their prostate weight was similar to that of Pten^(i)pe−/−^ mice (Supplementary Fig. 2C), but 50% of them presented cell infiltrates in the liver (Supplementary Fig. 2D). These infiltrates contained pan-cytokeratin positive cells surrounded by many CD45-positive cells (Fig. 2E). In contrast, mice with sarcomatoid tumors never presented such infiltrates. Moreover, as hepatic dissemination was never observed in Pten^(i)pe−/−^ mice at similar age, consistent with our previous analyses [13], p53 loss promotes the spread of *Pten*-deficient PECs. Altogether, these data demonstrate that tumors in DLP of Pten/Trp53^(i)pe−/−^ mice are more aggressive than those of Pten^(i)pe−/−^ mice.

As senescent cells secrete a cocktail of cytokines, chemokines, growth factors and matrix-remodeling factors, termed senescence-associated secretory phenotype (SASP), that might remodel their immune microenvironment and impact tumor progression [23], we immunophenotyped the prostates of control, Pten^(i)pe−/−^ and Pten/Trp53^(i)pe−/−^ mice 5 months AGI by flow cytometry. The abundance of immune cells (CD45^+^) was similar in Pten^(i)pe−/−^ and Pten/Trp53^(i)pe−/−^ mice, though higher than in control mice (Supplementary Fig. 3A). The number of myeloid cells [Ly6G^+^ neutrophils (Supplementary Fig. 3B) and CD11b^+^ macrophages (Supplementary Fig. 3C)], and of lymphoid populations [CD4^+^ and CD8^+^ T-cells] (Supplementary Fig. 3D–F) was similarly increased in both mutant mice. Note that 60% of CD8^+^ T-cells expressed the exhaustion marker PD-1 in both mouse models (Supplementary Fig. 3G). As Ly6G^+^ neutrophils are the major immune subpopulation of the DLP and the majority of CD8^+^ T lymphocytes express PD-1, the tumor microenvironment of both mutant mice has immunosuppressive characteristics.

### p53 deficiency promotes plasticity of *Pten*-null PECs via JAK/STAT3 signaling

**Fig. 3:**
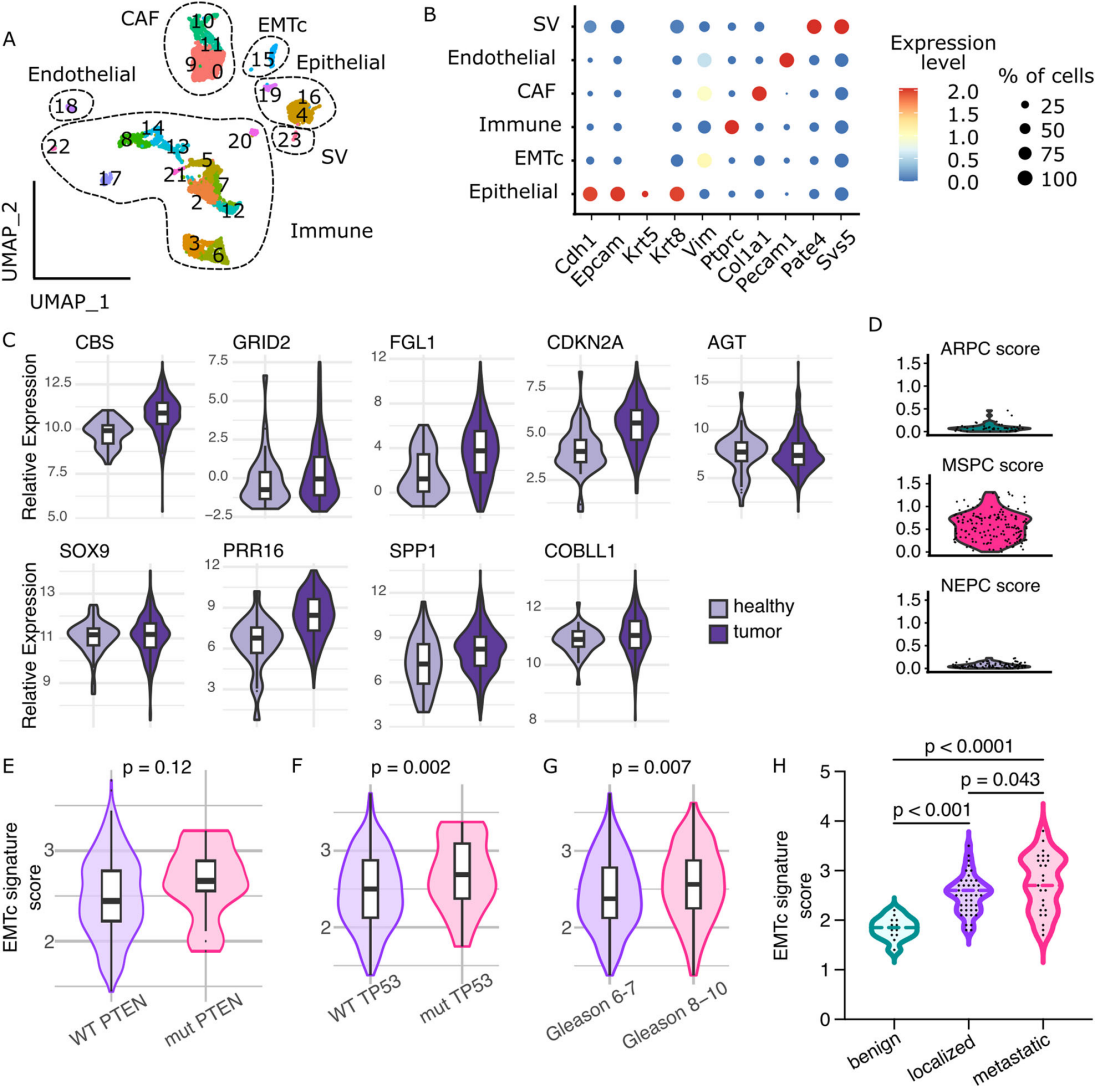
Single-cell transcriptomic analysis of Pten/Trp53^(i)pe−/−^ prostates. **A** Uniform Manifold Approximation and Projection (UMAP) of single cell transcriptomic analysis of 5449 cells from dissociated prostates of Pten/Trp53^(i)pe−/−^ mice 5 months AGI. **B** Dotplot of the average expression level of the marker genes used to annotate the cell populations, and the proportion of cell expressing them. **C** Violin plots of the 9 genes of the refined cluster 15 signature in prostate tumors and healthy tissue of the TCGA PRAD cohort. **D** Violin plots of the ARPC, MSPC and NEPC signature score described in Han et al. [6] in EMTc cells. **E–G** Violin plots of the score of the EMTc signature in patients with wild-type (WT) or mutated (mut) PTEN (**E**) or TP53 (**F**) and in patients with intermediate risk (Gleason score 6–7) or high risk (Gleason score 8–10) prostate cancer (**G**) in the TCGA PRAD cohort. Unpaired Student *T*-test. **H** Violin plots of the score of the EMTc signature in benign tissue, localized and metastatic primary prostate tumors of patients [20]. Multiple unpaired Student *T*-tests.

To investigate the molecular mechanisms underlying PIN progression, we performed single-cell RNA sequencing (scRNAseq) and recovered transcripts of 5449 cells isolated from 3 dissociated sarcomatoid-free prostates of Pten/Trp53^(i)pe−/−^ mice 5 months AGI. Annotation of the 23 identified cell clusters based on lineage markers revealed Immune (*Ptprc*, clusters 2, 3, 5, 6, 7, 8, 12, 13, 14, 17, 20, 21, 22), CAFs (*Vim, Col1a1*, clusters 0, 9, 10, 11), Endothelial (*Pecam1*, cluster 18), contaminant seminal vesicle (SV) (*Pate4*, cluster 23) and Epithelial (*Cdh1, Epcam*, clusters 4, 16 & 19) populations (Fig. 3A, B, Supplementary Table 1), the latter encompassing basal, luminal-B (LumB) [22] and luminal-C (LumC) cells (Supplementary Fig. 4A & B), as previously observed in Pten^(i)pe−/−^ mice [12]. However, one cluster (cluster 15) could not be annotated using lineage markers used in our previous analysis of Pten^(i)pe−/−^ mice. The signature of these cells, defined as globally distinguishing genes of this cluster that are upregulated in transcriptomes of tumors of the TCGA PRAD cohort (Fig. 3C, see “Methods”), was not detected in epithelial cells of Pten^(i)pe−/−^ mice 3, 6 or 9 months AGI [12] (Supplementary Fig. 4C). Interestingly, gene set enrichment analysis (GSEA) of cluster 15 signature revealed hallmarks of inflammatory and TGFß signaling, as well as of Epithelial-to-Mesenchymal Transition (EMT) [24] (Supplementary Fig. 4D, Supplementary Table 2). Cells of this cluster also expressed high levels of *Cdkn2a* (p16/p19) and of *Hoxb13* encoding a lineage marker conserved throughout prostate cancer progression [25] (Supplementary Fig. 4E), indicating their luminal epithelial origin. This cluster was thus annotated as EMT cells (EMTc).

Recently, 3 transcriptional subtypes of human prostate cancer were reported: (i) androgen receptor pathway-positive prostate cancer (ARPC), (ii) mesenchymal and stem-like prostate cancer (MSPC) and (iii) neuroendocrine prostate cancer (NEPC) [6]. Interestingly, EMTc cells have a much higher signature score of MSPC than of ARPC and NEPC (Fig. 3D). In addition, while the EMTc signature score (Fig. 3C) was similar in tumors of prostate cancer patients with wild-type or mutated PTEN, it was higher in tumors of patients with mutated TP53 than of those with wild-type TP53 in the TCGA PRAD cohort (Fig. 3E, F). In addition, the score of this signature was higher in patients with high-risk disease (Gleason score 8–10) (Fig. 3G). Finally, the EMTc signature score was higher in patients with localized disease than in benign prostates, and it was even higher in patients with metastases in the dataset reported by Grasso et al. [20]. (Fig. 3H). Together, our data uncover a subset of luminal cells in murine *Pten*- and *Trp53*-deficient tumors that shares transcriptomic features with human prostate tumors exhibiting increased mesenchymal and stem-like characteristics and metastasis.

**Fig. 4:**
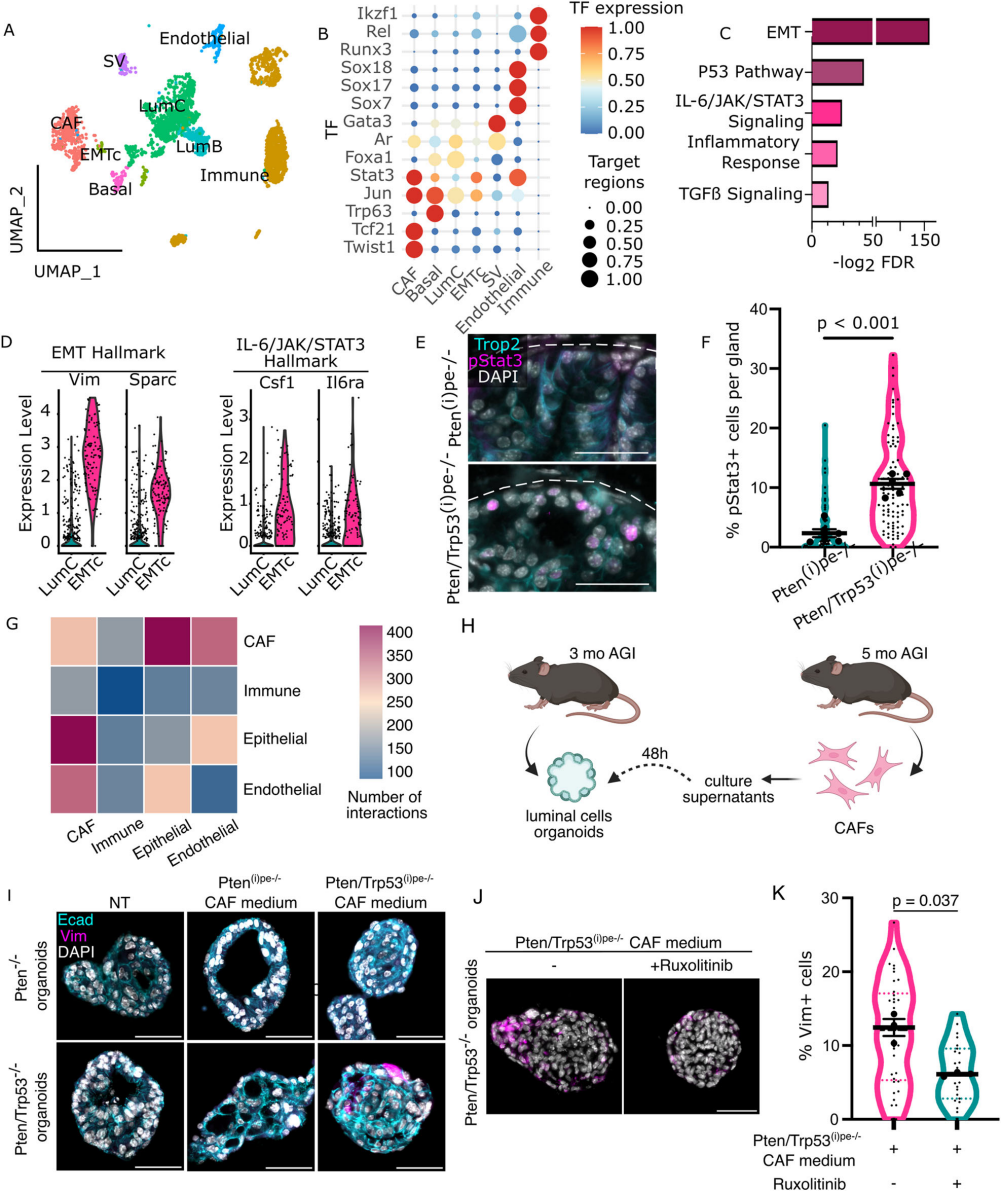
Characterisation of the signalling driving epithelial cell plasticity in prostatic tumors of Pten/Trp53^(i)pe−/−^ mice. **A** UMAP of Single-nuclei Assay for Transposase-Accessible Chromatin (ATAC) sequencing of 3973 cells from dissociated DLP of Pten/Trp53^(i)pe−/−^ mice 5 months AGI. **B** Heatmap representing the eRegulons specific to the cell populations identified by SCENIC + . Color scale represents the transcript levels of the activator transcription factors (TF), and the dot size the enrichment of their motifs in open chromatin regions. **C** Gene Set Enrichment Analysis (GSEA) of transcripts upregulated in EMTc compared to LumC cells. **D** Violin plots of the transcript levels of the indicated genes selected from EMT and IL6/Jak/Stat3 hallmarks identified in (**C**), in LumC cells and EMTc. **E** Representative immunofluorescent staining of pStat3 (magenta) and Trop2 (cyan) in DLP sections from Pten^(i)pe−/−^ and Pten/Trp53^(i)pe−/−^ mice 5 months AGI. Nuclei are stained with DAPI (white). Scale bar: 50 µm. *n* = 4 mice per genotype. Dashed line shows the limit of the gland. **F** Quantification of pStat3-positive cells located within less than 30 µm from the basal lamina of glands in DLP of Pten^(i)pe−/−^ and Pten/Trp53^(i)pe−/−^ mice 5 months AGI. *n* ≥ 5 mice per group. Unpaired Student *t*-test. **G** Heatmap representing the number of interactions between the indicated cell populations in prostates of Pten/Trp53^(i)pe−/−^ prostates 5 months AGI, determined by CellPhoneDB [21] analysis. **H** Schematic representation of the experimental procedure to evaluate the effect of culture media of CAFs isolated from mice 5 months AGI on organoids derived from DLPs of mutant mice 3 months AGI. **I** Representative immunofluorescent staining of E-cadherin (Ecad, cyan) and Vimentin (Vim, magenta) in Pten^−/−^ and Pten/Trp53^−/−^ organoids, treated or not (NT) with supernatants from cultured CAFs isolated from prostates of Pten^(i)pe−/−^ or Pten/Trp53^(i)pe−/−^ mice 5 months AGI. Nuclei are stained with DAPI (white). *n* = 3 independent experiments. Scale bar: 50 µm. **J**, **K** Representative immunofluorescent staining of Vimentin (Vim, magenta) (**J**) and quantification of Vim positive cells (**K**) in Pten/Trp53^−/−^ organoids, treated with supernatants from cultured CAFs isolated from prostates of Pten/Trp53^(i)pe−/−^ mice 5 months AGI, supplemented or not with Ruxolitinib (10 µM). Nuclei are stained with DAPI (white). *n* = 3 independent experiments. Scale bar: 50 µm. Unpaired Student *t*-test.

To further characterize the cell populations present in the DLP of sarcomatoid-free Pten/Trp53^(i)pe−/−^ mice 5 months AGI, we performed single-nuclei Assay for Transposase-Accessible Chromatin sequencing (snATACseq). Cluster annotation identified similar cell populations than those obtained by scRNAseq, namely Immune (clusters 2, 17, 5, 19, 20, 16), Endothelial (cluster 9), CAF populations (clusters 0, 14, 18), Epithelial subpopulations [LumC (clusters 3, 8, 1, 4, 10), LumB (cluster 7), Basal (cluster 13)], SV (cluster 11)] and, importantly, EMTc (cluster 15) (Fig. 4A, Supplementary Table 3). To identify candidate factors driving cell-specific gene expression, we performed single-cell multiomic inference of enhancers and gene regulatory networks (SCENIC + ) analyses [26] by integrating chromatin accessibility and gene expression profiles obtained from snATACseq (Fig. 4A) and scRNAseq (Fig. 3A) datasets, respectively. This analysis revealed eRegulons of Runx3, Rel and Izkf1 selectively in the immune populations, of Sox7, Sox17 and Sox18 selectively in the endothelial cluster, and of Twist1 and Tcf21 selectively in the CAF populations (Fig. 4B & Supplementary Table 4). In addition, within epithelial subpopulations, the eRegulon of Trp63 was selective to the basal cluster, whereas Foxa1 and Androgen receptor (Ar) eRegulons were enriched in the LumC cluster. In contrast, Foxa1 and Ar eRegulons were low in the EMTc. Moreover, Stat3 expression and its target site accessibility were higher in EMTc than in LumC cells. Interestingly, GSEA of differentially expressed genes (DEGs) between EMTc and LumC cells revealed an enrichment of epithelial-mesenchymal transition (e.g. *Vim* and *Sparc*), as well as IL6/Jak/Stat3 signaling-related genes (e.g. *Csf1 and Il6ra)* (Fig. 4C, D, and Supplementary Tables 5 & 6). Importantly, immunofluorescent analyses showed that pStat3 (T705)-positive PECs were much more abundant in PINs of Pten/Trp53^(i)pe−/−^ mice than of Pten^(i)pe−/−^ mice 5 months AGI, and were mainly located near the basal lamina, in proximity to the stroma (Fig. 4E, F). Thus, Stat3 signaling induced in *Pten-* and *Trp53*-deficient PECs might require signals from the stroma to induce their plasticity.

### IL-6 produced by cancer-associated fibroblasts induces plasticity of PTEN- and p53-deficient PECs

To identify cell types and molecular factors that might promote Stat3 signaling in PECs of Pten/Trp53^(i)pe−/−^ mice, we interrogated the scRNAseq data using CellPhoneDB [27]. These analyses predicted that epithelial populations interact mostly with CAFs (Fig. 4G, Supplementary Table 7). To determine whether CAFs from prostates of mutant mice secrete factors that promote PEC plasticity, Pten^−/−^ and Pten/Trp53^−/−^ organoids were treated for 48 h with the supernatants of CAFs isolated from prostates of Pten^(i)pe−/−^ or Pten/Trp53^(i)pe−/−^ mice 5 months AGI (Fig. 4H). Whereas all cells in organoids from both lines were E-cadherin-positive, some also expressed Vimentin (Vim) in Pten/Trp53^−/−^ organoids treated with supernatants of CAFs isolated from prostates of Pten/Trp53^(i)pe−/−^ mice, but not of Pten^(i)pe−/−^ones (Fig. 4I). Importantly, co-treatment of Pten/Trp53^pe−/−^ organoids with Pten/Trp53^(i)pe−/−^ CAF supernatants and the JAK kinase inhibitor Ruxolitinib reduced the proportion of Vim^+^ cells (Fig. 4J, K), demonstrating the driver role of Jak/Stat signaling in plasticity induction. In contrast, no Vim^+^ cells were detected in Pten^−/−^ organoids treated with supernatants of CAFs isolated from prostates of Pten ^−/−^ or Pten/Trp53^−/−^ mice, indicating a crosstalk between PECs and CAFs selectively in Pten/Trp53^(i)pe−/−^ tumors.

**Fig. 5:**
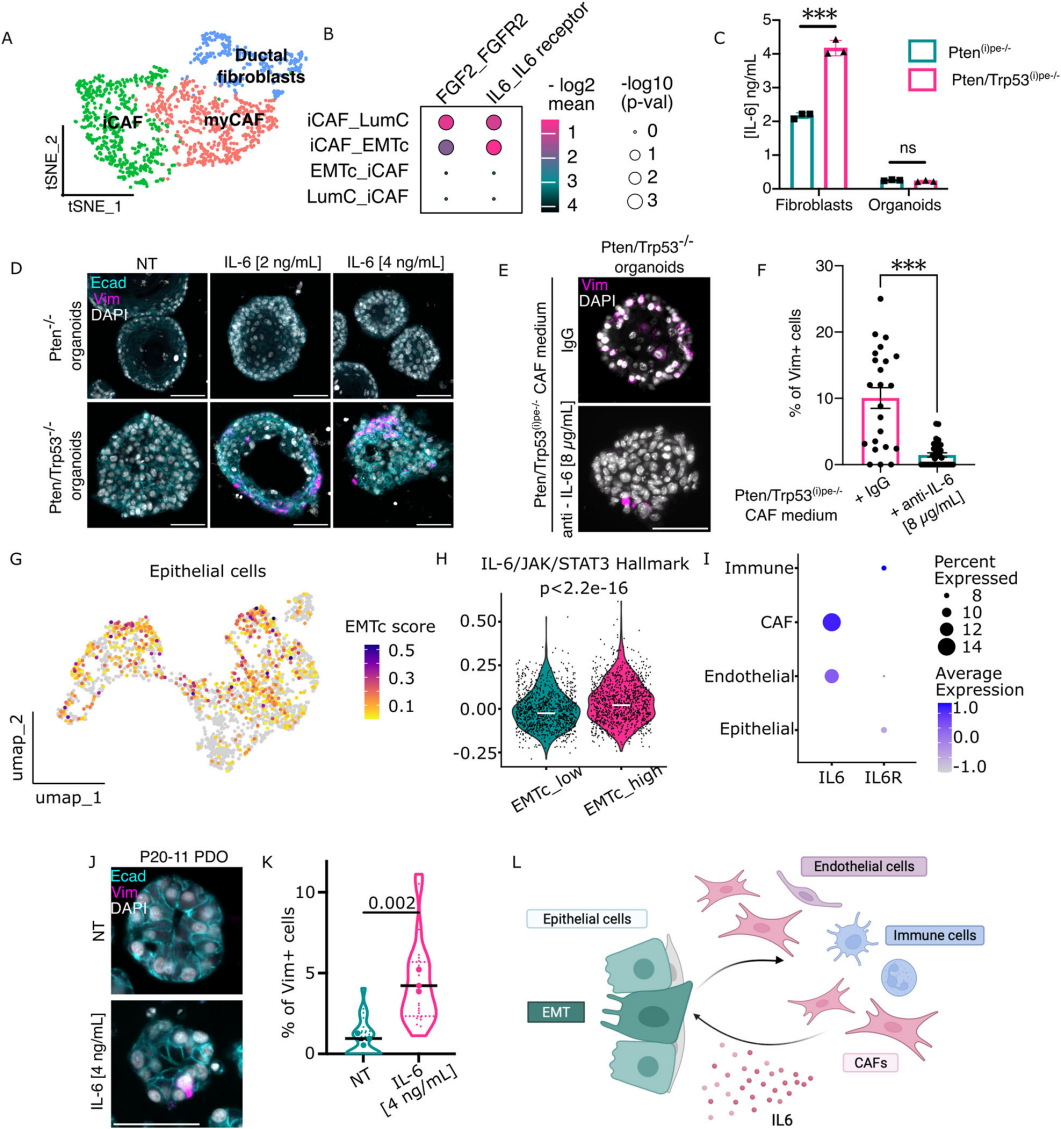
Characterization of the crosstalk between epithelial and stromal cells in prostatic tumors of Pten/Trp53^(i)pe−/−^ mice. **A** t-SNE of fibroblast subpopulations in prostates of Pten/Trp53^(i)pe−/−^ mice 5 months AGI. **B** Most significant unilateral interactions of the indicated secreted signaling between iCAFs and EMTc, or iCAFs and LumC cells. The first cell population in the interacting couple (interactant1_interactant2) expresses the ligand, and the second the receptor (ligand_receptor). **C** IL-6 levels in culture supernatants of Pten^−/−^ and Pten/Trp53^−/−^organoids and fibroblasts isolated from prostates of Pten^(i)pe−/−^ and Pten/Trp53^(i)pe−/−^ mice, 5 months AGI. *n* = 3 mice per group. ns non-significant, *p* ≥ 0.05; ***p < 0.001. Two-way ANOVA followed by Tukey’s post-hoc. **D** Representative immunofluorescent staining of E-cadherin (Ecad, cyan) and Vimentin (Vim, magenta) in Pten^−/−^ and Pten/Trp53^−/−^ organoids, treated or not (NT) with IL-6 at 2 and 4 ng/mL. Nuclei are stained with DAPI (white). Scale bar: 50 µm. *n* = 3 independent experiments. **E**, **F** Representative immunofluorescent staining of Vimentin (Vim, magenta) (**E**) and quantification of Vim positive cells (**F**) in Pten/Trp53^−/−^ organoids, treated with supernatants from cultured CAFs isolated from prostates of Pten/Trp53^(i)pe−/−^ mice 5 months AGI, supplemented with control IgG or IL-6 neutralizing antibodies. Nuclei are stained with DAPI (white). *n* = 3 independent experiments. Scale bar: 50 µm. Mann-Whitney test. **G** EMTc signature score in epithelial cells from IDC-containing prostate tissue from patients in the dataset of Wong et al. [32]. **H** Violin plot of the score of the IL6/JAK/STAT3 Hallmark in epithelial cells with a low (below median) and a high (above median) EMTc signature score. **I** Dotplot of the average expression level of the indicated genes in cell populations reported by Wong et al. [32], and the proportion of cell expressing them. **J**, **K** Representative immunofluorescent staining of Vimentin (Vim, magenta) and E-cadherin (Ecad, cyan) (**J**) and quantification of Vim positive cells (**K**) in P20-11 patient-derived organoids, treated or not (NT) with IL-6 at 4 ng/mL. Nuclei are stained with DAPI (white). Scale bar: 50 µm. *n* = 3 independent experiments. Unpaired *t*-test. **L** Schematic representation of the crosstalk between PECs and stromal cells stimulating CAFs to produce IL-6 which in turn activates JAK/STAT3 signaling in PECs that promotes EMT.

Molecules secreted by CAFs might contribute to tumor progression, invasion and immune escape [28]. CAF subclustering revealed 3 populations that were annotated based on the expression of previously reported selective marker genes [29–31]: ductal fibroblasts (*Wnt2, Rorb*), myofibroblastic CAFs (“myCAFs” [*Acta2, Tagln, Mmp11, Myl9, Postn, Tpm1, Tpm2]*), and inflammatory CAFs (“iCAFs” [*Il6, Cxcl12, Dpt, Has1, Cxcl1, Ccl2*]) (Fig. 5A). As expected, GSEA of their transcriptomic signatures revealed an enrichment of pathways related to extracellular matrix organization in myCAF and ductal fibroblasts, and immune system activation in iCAFs (Supplementary Fig. 4F). Given the IL-6/JAK/STAT3 signaling enrichment in EMTc compared to LumC cells (Fig. 4C), we interrogated the predicted interactions through secreted molecules between iCAFs and LumC cells or EMTc. The most significant and specific interaction was between *Il6* expressed by iCAFs and IL-6 receptor (*Il6ra*) of EMTc (Fig. 5B & Supplementary Table 8). Moreover, whereas culture supernatants of Pten^−/−^ and Pten/Trp53^−/−^ organoids contained low IL-6 levels, those of CAFs from Pten^(i)pe−/−^ mice and Pten/Trp53^(i)pe−/−^ ones contained 10-fold and 20-fold higher IL-6 levels, respectively (Fig. 5C). Importantly, IL-6 induced Vimentin expression in a dose-dependent manner in Pten/Trp53^−/−^ organoids, but not in Pten^−/−^ ones (Fig. 5D). Furthermore, IL-6 neutralizing antibodies strongly decreased the number of Vimentin-positive cells in *Pten*- and *Trp53*-deficient organoids induced by Pten/Trp53^(i)pe−/−^ CAF supernatants (Fig. 5E, F).

Analyses of the single-cell transcriptomic dataset of Wong et al. [32] revealed that epithelial cells of IDC in patients expressed EMTc signature genes (Fig. 5G) and that the IL6/JAK/STAT3 signaling pathway Hallmark was higher in cells with a high EMTc score (Fig. 5H). Importantly, *IL6* transcripts were detected only in CAFs and endothelial cells of those tumors, whereas those of IL-6 receptor, *IL6R*, were mostly detected in epithelial and immune cells (Fig. 5I). In addition, IL-6 treatment of the *PTEN* and *TP53*-deficient patient-derived organoid line P20-11 [18] induced Vimentin expression (Fig. 5J, K). Together, these results show that *Trp53* deficiency in PECs enhances IL-6 production in CAFs, which in turn promotes Jak/Stat-induced epithelial cell plasticity (Fig. 5L).

## Discussion

*PTEN* and *TP53* genomic alterations are frequent in advanced prostate tumors and are associated with prostate cancer aggressivity. However, as some occur in preneoplastic lesions, and as the impact of combined *PTEN* and *TP53* loss in PECs on their progression is poorly characterized, we compared prostate tumorigenesis in Pten/Trp53^(i)pe−/−^ and Pten^(i)pe−/−^ mice, in which *Trp53* and/or *Pten* are inactivated in prostatic luminal cells at adulthood. Moreover, we addressed the clinical relevance of our findings by interrogating human prostate cancer datasets.

We show that *Trp53* and/or *Pten* are efficiently inactivated in luminal cells of the DLP of adult Pten/Trp53^(i)pe−/−^ and Pten^(i)pe−/−^ mice, respectively, and that PECs actively proliferate between 1 and 3 months AGI, leading to PINs at high incidence in these prostatic lobes of both mutant mice, in agreement with our previous study [14]. Moreover, we demonstrate that both *Pten*- and *Pten/Trp53*-null PECs are growth arrested at a later time and are SA-ß-gal-positive. However, whereas PECs of Pten^(i)pe−/−^ mice express the cell cycle inhibitors p21 and p16, those of Pten/Trp53^(i)pe−/−^ mice only express p16. Thus, both *Pten*- and *Pten/Trp53*-deficient PECs become senescent in DLP after the proliferation phase, but their senescence state might be different. Chen and colleagues reported the development of large prostatic tumors in the anterior prostate of Pten^pc−/−^/Trp53^pc−/−^ mice, in which the tumor suppressor genes are inactivated in PECs before puberty [15]. As no SA-ß-gal activity was observed in such *Pten*/*Trp53*-deficient PECs in contrast to those of aged-matched Pten^pc−/−^ mice, it was concluded that senescence of *Pten*-deficient tumors is p53-mediated. Even though PSA-CreER^T2^-mediated gene inactivation is less efficient in the AP than in the DLP [13], and tumor formation is heterogeneous in this lobe, we detected p16-positive cells in PINs, and observed large sarcomatoid tumors in the AP in about 20% of Pten/Trp53^(i)pe−/−^ mice between 5 and 7 months AGI. Taken together, these data show that *Pten*– deficient PECs enter senescence in the absence of p53 in the DLP, and highlight lobe-specific tumor progression.

Importantly, our multiomic single-cell analyses revealed that combined loss of PTEN and p53 leads to an emergence of a subset of luminal cells during the senescent phase, termed EMTc, characterized by an epithelial-to-mesenchymal transition signature. Further investigation of tumor progression in the DLP, shown to resemble human prostate peripheral zone where most adenocarcinoma develop [33], revealed that Pten/Trp53^(i)pe−/−^ mice present more invasive adenocarcinoma than Pten^(i)pe−/−^ mice, as well as cell infiltrates in the liver at a 50% penetrance. Importantly, such infiltrates were never observed in Pten/Trp53^(i)pe−/−^ mice with sarcomatoid tumors, nor in Pten^(i)pe−/−^ mice at similar age, indicating that metastatic spreading selectively originates from *Pten/Trp53*-null adenocarcinoma. These results are in line with previous observations in breast tumors, showing that those with a partial EMT state display a higher metastatic capacity than fully dedifferentiated ones [34], and in prostate cancer mouse models showing that KRas overexpression in Pten-null PECs promotes intermediate epithelial-mesenchymal states with high tumoroid-forming capacity and metastasis to soft tissues [35, 36].

Our single-cell analyses revealed that EMTc are characterized by an induction of Jak/Stat3 signaling and a strong *Cdkn2a* (p16/p19) signature. Importantly, we demonstrate that tumors of patients with high-risk prostate cancer and with metastatic disease have a high EMTc signature score. In addition, EMTc share a transcriptomic signature with the recently identified human mesenchymal and stem-like prostate cancer (MSPC) subtype, that is present in treatment-naïve patients, enriched in metastatic prostate cancer, and frequently mutated for *PTEN* and *TP53* [6]. Moreover, as patients with MSPC are poor responders to androgen receptor signaling inhibitors, impairing the emergence of this cell population represents an attractive strategy to prevent tumor progression.

Previous studies reported that IL6/Jak/Stat3 pathways is operating at multiple levels during prostate tumor progression. Pencik et al. showed an increased aggressivity of prostate tumors in Pten^pc−/−^/Stat3^pc−/−^ mice, with a concomitant deregulation of the senescent state by disrupting the ARF–MDM2–p53 tumor suppressor axis [37]. In contrast, Toso et al. showed decreased prostate cancer progression in Pten^pc−/−^/Stat3^pc−/−^ mice, promoted by clearance of senescent cells by the immune microenvironment [38]. Furthermore, Nowak et al. reported IL-6 production in PECs of Pten^pc−/−^/Trp53^pc−/−^ mice leading to an autocrine/paracrine STAT3 activation in PECs and CAFs, promoting MYC activation and metastasis [39]. In addition, CAF-produced IL-6 was shown to attenuate p53-mediated response to chemotherapy in LNCaP cells [11]. Moreover, Trp53/Rb1-co-deletion in prostatic epithelial cells induced Jak/Stat signaling and cell plasticity in a cell-autonomous manner leading to the progression of prostate tumors towards neuroendocrine prostate cancer and resistance to androgen receptor signaling inhibitors [40, 41]. However, the potential impact of the TME on the induction of PTEN- and p53-deficient PECs plasticity was unknown.

We demonstrate that IL-6 induces the expression of the mesenchymal marker Vimentin in PECs of Pten/Trp53^(i)pe−/−^ mice, but not of Pten^(i)pe−/−^ ones. Intriguingly, the number of pStat3-positive cells was much higher in DLP of Pten/Trp53^(i)pe−/−^ mice than of Pten^(i)pe−/−^ ones, and these cells were preferentially located in the vicinity of stromal cells, indicating that EMTc might be instructed by paracrine signals of the microenvironment. Importantly, we show that CAFs produce at least 10 times more IL-6 than organoids from PECs of both mouse models, and that CAFs from prostates of Pten/Trp53^(i)pe−/−^ mice produce twice as much IL-6 than those of Pten^(i)pe−/−^ ones, indicating that signals from Trp53-deficient tumors stimulate IL-6 secretion by CAFs. Moreover, as culture supernatants of CAFs from Pten/Trp53^(i)pe−/−^ mice but not from Pten^(i)pe−/−^ ones induced Vimentin expression only in Pten/Trp53^−/−^ PECs, and as this effect was dampened by Jak/Stat signaling inhibition and by IL-6 neutralizing antibodies, we conclude that epithelial cell plasticity is driven by a feedforward loop, involving CAF-produced IL-6 promoting Jak/Stat3 pathway in PECs located in proximity of the TME, in contrast to an autocrine/paracrine epithelial signaling proposed by previous studies [39]. Whether PECs act directly on CAFs to enhance IL-6 or through interactions with other cells (e.g. immune and endothelial cells) present in the TME requires further investigations.

## Conclusion

Taken together, our study uncovers an epithelial population with partial EMT characteristics emerging selectively in PTEN- and p53-deficient senescent PINs. We provide evidence that this cell population characterized by enhanced JAK/STAT3 signaling is induced by IL-6 produced by CAFs within the immune microenvironment containing immunosuppressive myeloid cells and exhausted lymphocytes. Importantly, as the presence of the EMTc cells correlates with metastatic dissemination, the identified crosstalk represents an attractive therapeutic target.

## Supplementary information

Figure S1

Figure S2

Figure S3

Figure S4

Figure S5

Dataset 1

## Data Availability

All data needed to evaluate the conclusions in the paper are present in the paper and/or the Supplementary Materials. Sequencing data are deposited at the Gene Expression Omnibus (GEO) database (GSE236042). Code for scRNAseq and snATACseq analysis is available at Github repository on demand.
