# Supplementary figures and images for: p53-loss induced prostatic epithelial cell plasticity and invasion is driven by a crosstalk with the tumor microenvironment
Source: Cell Death Dis. 2025 Jan 26;16(1):46. doi: 10.1038/s41419-025-07361-1 (PMC11770131; doi:10.1038/s41419-025-07361-1)

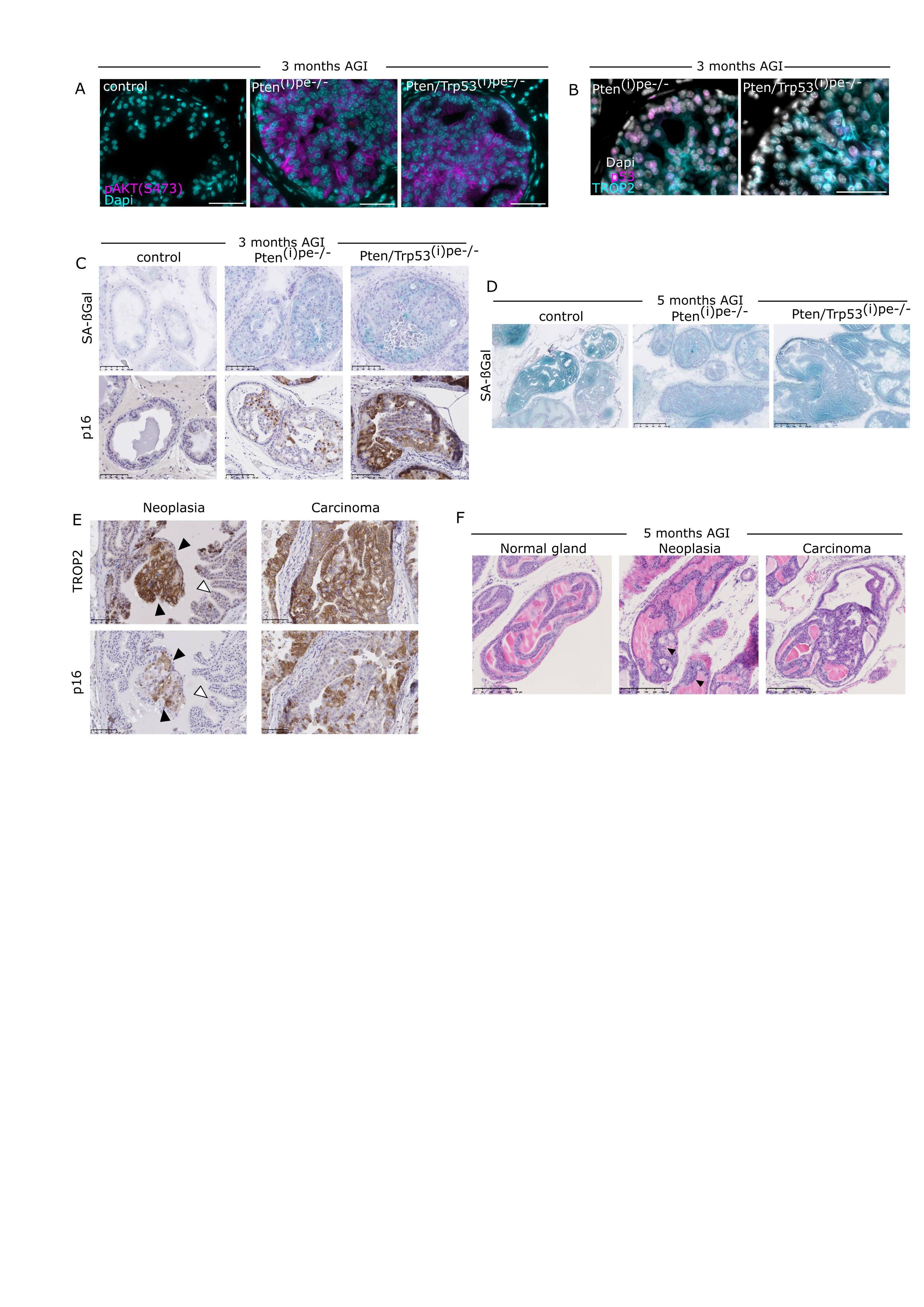

Supplement: Supplementary file 1 — Figure S1 [file 41419_2025_7361_MOESM1_ESM.png]

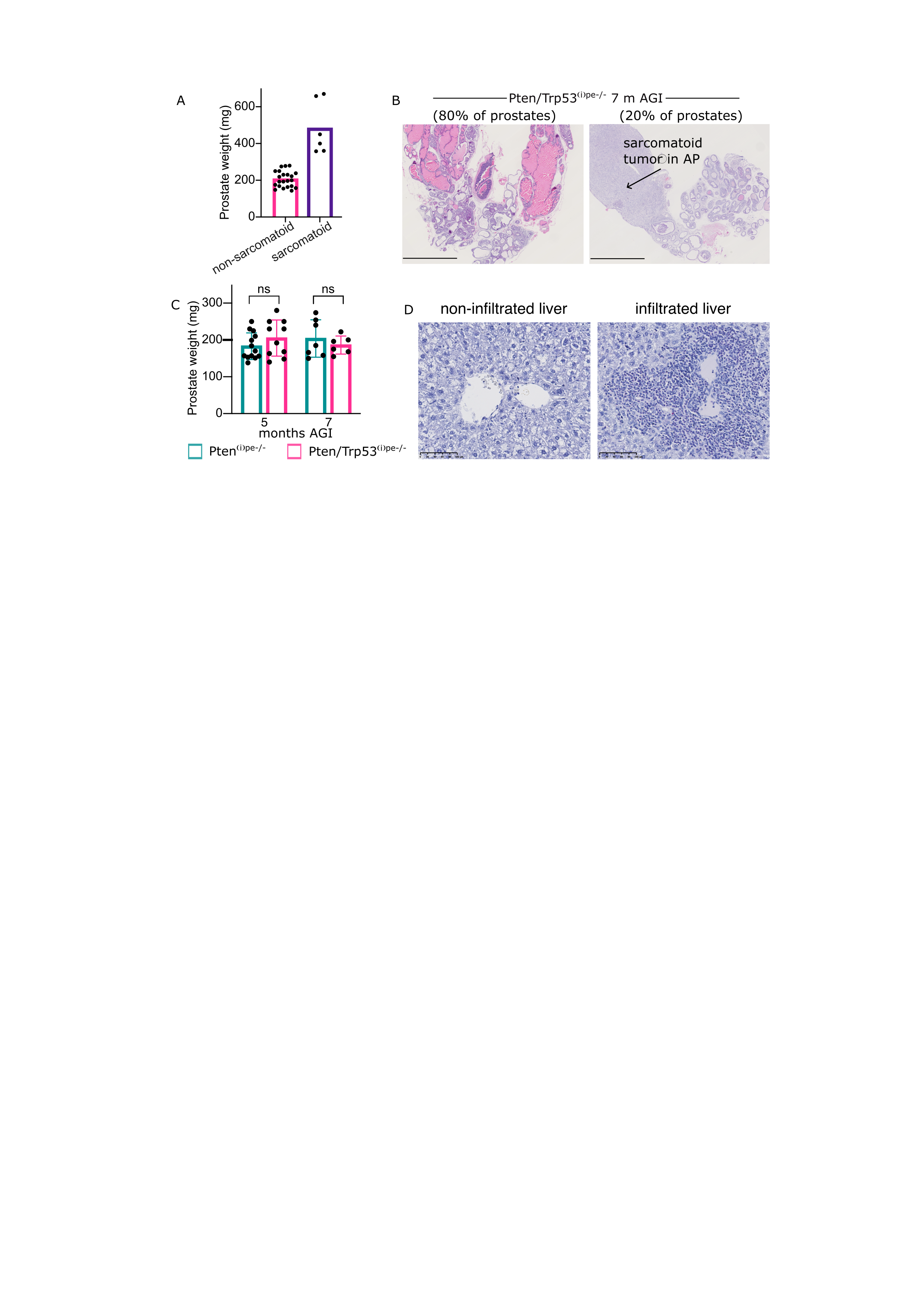

Supplement: Supplementary file 2 — Figure S2 [file 41419_2025_7361_MOESM2_ESM.png]

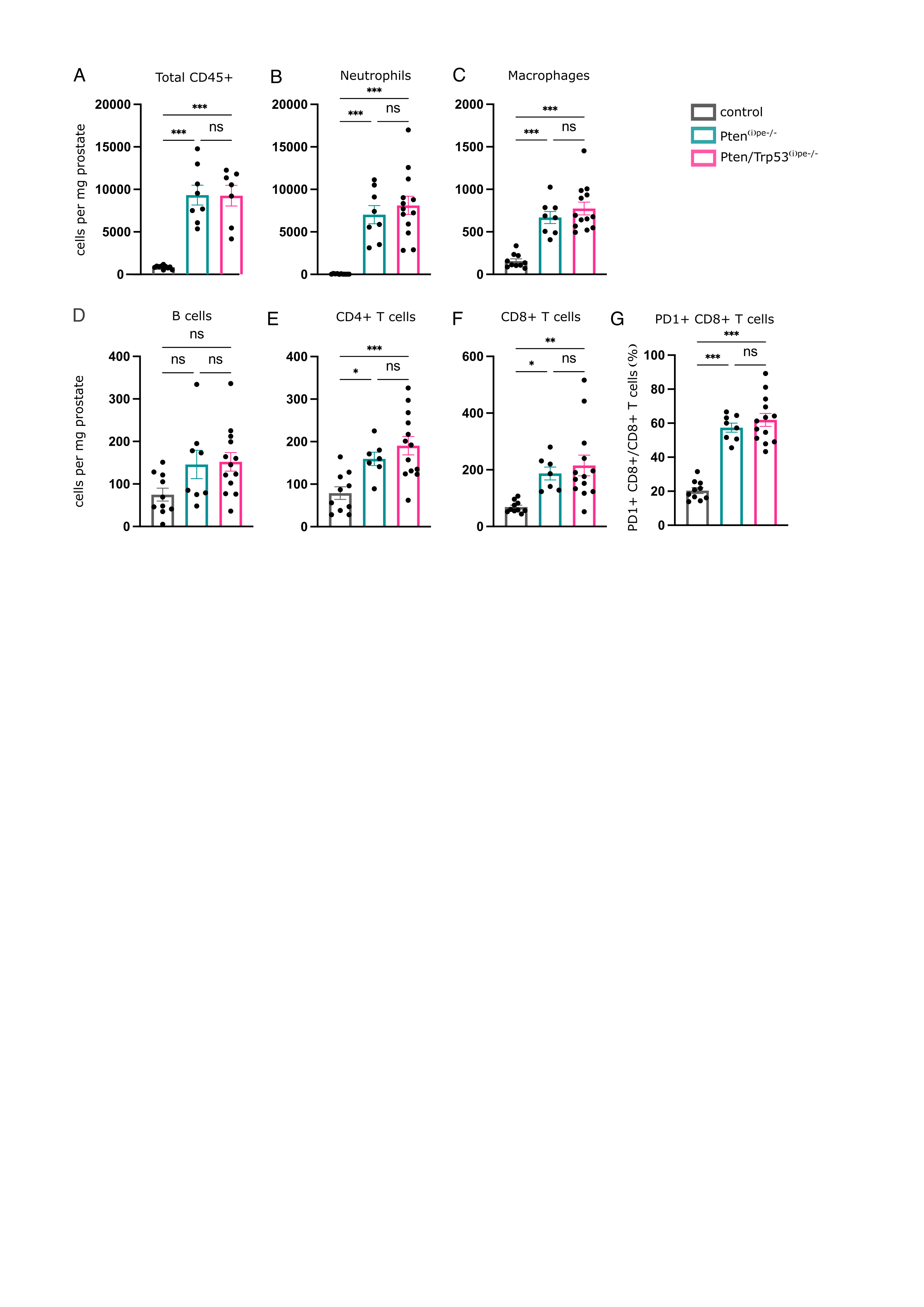

Supplement: Supplementary file 3 — Figure S3 [file 41419_2025_7361_MOESM3_ESM.png]

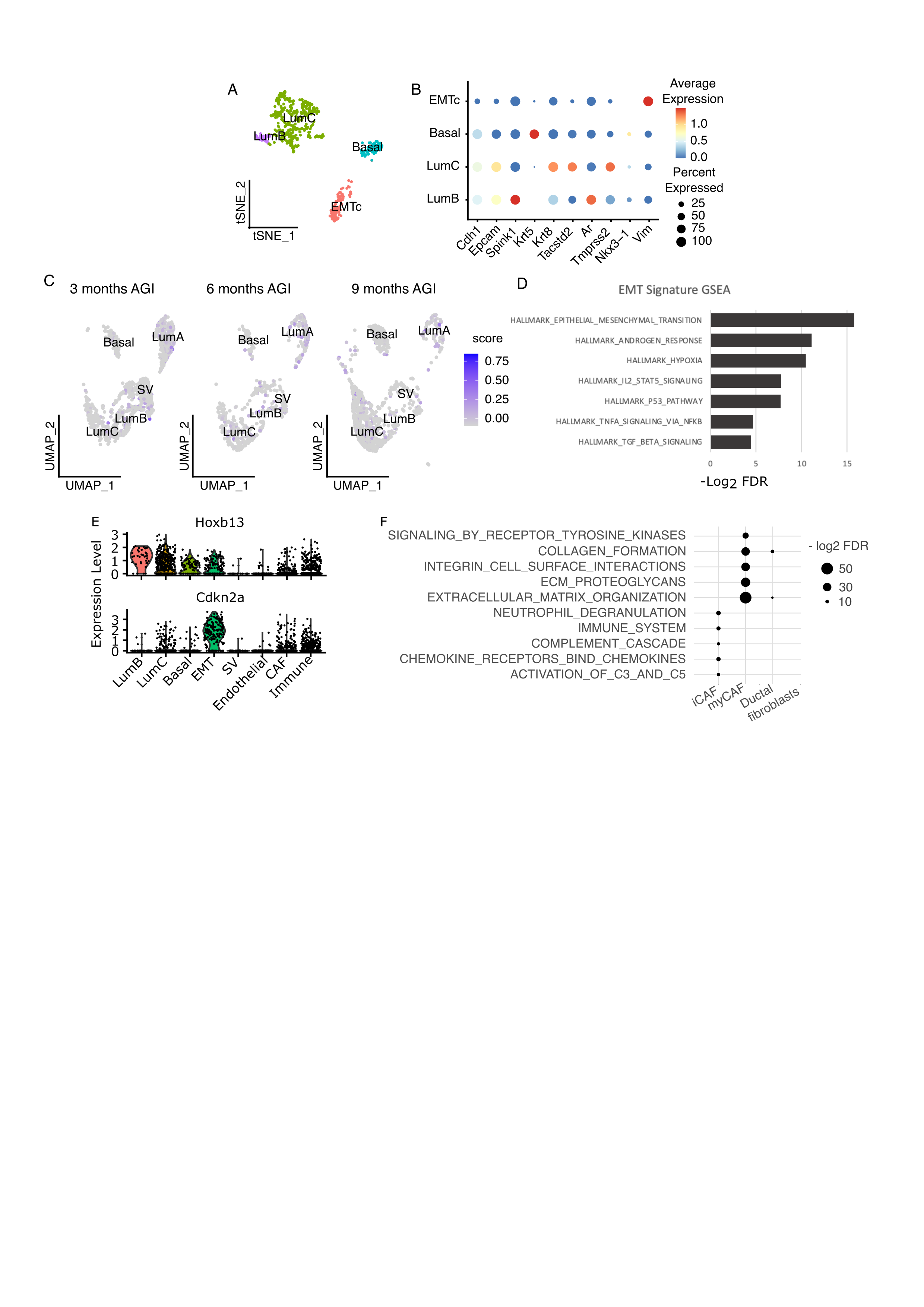

Supplement: Supplementary file 4 — Figure S4 [file 41419_2025_7361_MOESM4_ESM.png]

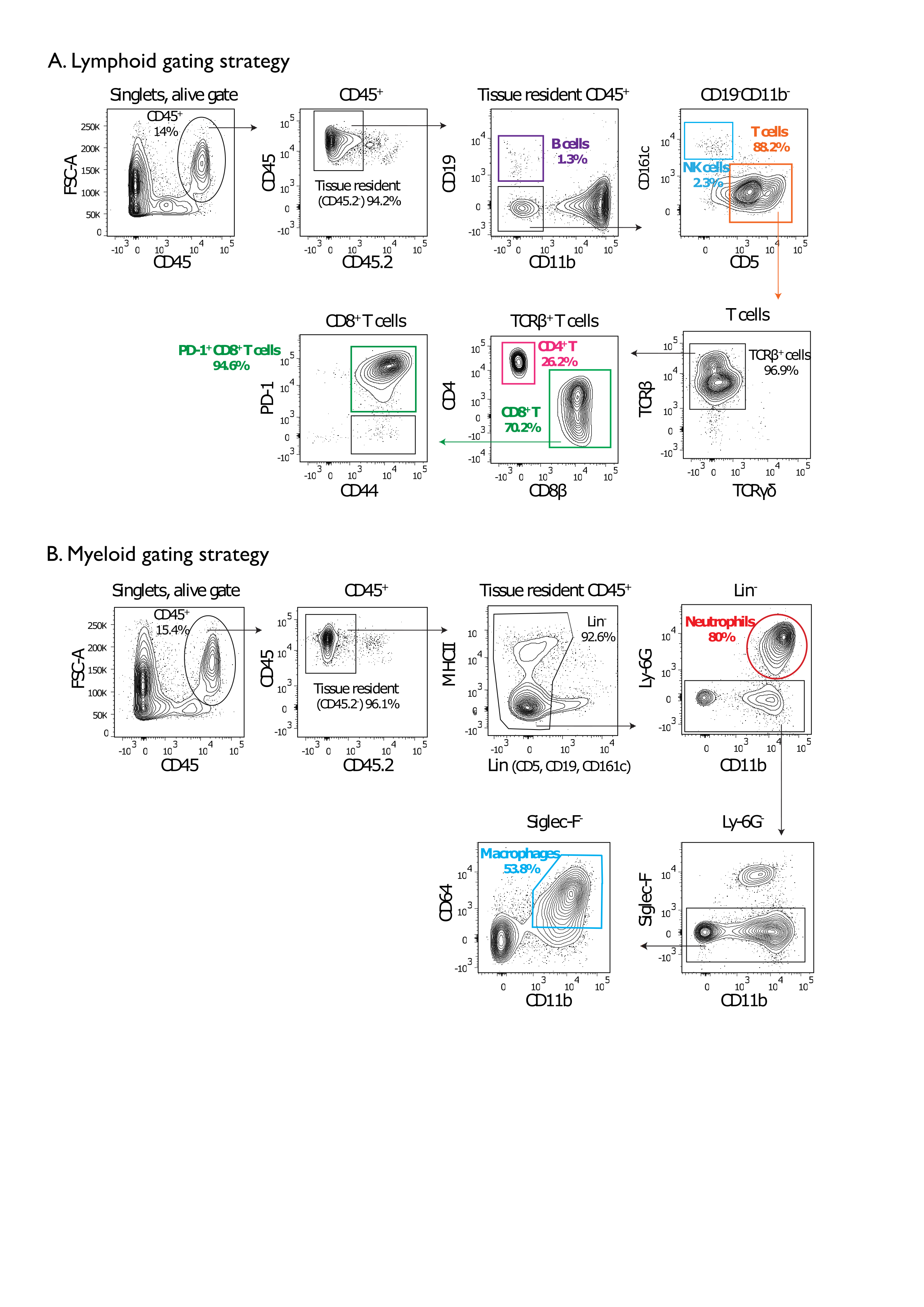

Supplement: Supplementary file 5 — Figure S5 [file 41419_2025_7361_MOESM5_ESM.png]
